# Clinical presentation of asymptomatic and symptomatic women who tested positive for genital gonorrhoea at a sexual health service in Melbourne, Australia

**DOI:** 10.1017/S0950268820002265

**Published:** 2020-09-28

**Authors:** Mario Martín-Sánchez, Christopher K. Fairley, Jason J. Ong, Kate Maddaford, Marcus Y. Chen, Deborah A. Williamson, Catriona S. Bradshaw, Eric P.F. Chow

**Affiliations:** 1 Melbourne Sexual Health Centre, Alfred Health, Melbourne, VIC, Australia; 2 Preventive Medicine and Public Health Training Unit PSMar-UPF-ASPB, Barcelona, Spain; 3 Central Clinical School, Monash University, Melbourne, VIC, Australia; 4 Department of Microbiology and Immunology, Microbiological Diagnostic Unit Public Health Laboratory, The University of Melbourne at The Peter Doherty Institute for Infection and Immunity, Melbourne, VIC, Australia; 5 Centre for Epidemiology and Biostatistics, Melbourne School of Population and Global Health, The University of Melbourne, Melbourne, Australia

**Keywords:** Asymptomatic infections, *Neisseria gonorrhoeae*, sexual behaviours, sexually transmitted infections, women

## Abstract

Gonorrhoea cases in women have been rising in Australia in the 2010s but the cause of the increase is not well understood. This cross-sectional study aimed to describe the characteristics of genital gonorrhoea infection in women attending the Melbourne Sexual Health Centre, Australia. Gonorrhoea cases were diagnosed by nucleic acid amplification test (NAAT) and/or culture. Genitourinary specimens were obtained in 12 869 clinic visits in women aged 16 years or above between August 2017 and August 2018. Genital gonorrhoea was detected in 142 (1.1%) of the visits. Almost half of the cases were asymptomatic, 47.9% [95% confidence interval (CI) 39.8–56.1%]; yellow, green or pus-like vaginal discharge was present in 11.3% (95% CI 7.0–17.6%) and other genital symptoms in 40.8% (95% CI 33.1–49.1%) of the cases. The mean time between last sexual contact and onset of symptoms was 7.3 days and between the onset of symptoms to presentation to the clinic was 12.1 days. Half of the cases of genital gonorrhoea among women are asymptomatic and these cases would have been missed by testing of only symptomatic women. Further epidemiological and behavioural research is required to understand the temporal changes in sexual practices among women in Australia.

## 1. Introduction

It is considered that the current epidemic of gonorrhoea mainly affects men, particularly gay, bisexual and other men who have sex with men (MSM) in Australia [1, 2]. Gonorrhoea is deemed to be uncommon among women in major Australian cities (<1% in the population); however rises have been observed in the 2010s [3–5]. This increase has also been reported among women in other high-income countries such as England, Spain or the USA [6–10]. The rise in gonorrhoea cases has also been observed in MSM and heterosexual men [11]. Overall, there was an 80% increase in the notification rate of gonorrhoea (from 65.5 to 118.0 per 100 000) between 2013 and 2017 in the total Australian population.

The reasons underlying the rise in gonorrhoea among women remain unclear. While the Australian Study of Health and Relationships surveys reported there was no change in condomless penile-vaginal sex nor in the number of sexual partners among women between 2001 and 2013 [12, 13], trend data on sexual practices among Australian women thereafter is scarce and other factors (e.g. sex overseas, use of smartphone dating applications) may have changed over time and contributed to the rise of gonorrhoea [3, 14–16]. Other reasons besides sexual practices may include sociodemographic changes or changes in health services use. The increase in health care demand from the rising rates of sexually transmitted infections (STIs) may be making it harder to access health care, and consequently increasing the duration of untreated infection which would lead to ongoing transmission [17–19].

Genital gonorrhoea can be asymptomatic in women and, hence individuals may not be aware of their infection for long periods. Untreated gonorrhoea in women can lead to pelvic inflammatory diseases and other serious complications including ectopic pregnancy or infertility [20], making prompt identification and treatment especially important. However, recent studies on genital gonorrhoea symptomatology in women are very limited and most studies were conducted in the 1970s and culture was used for gonorrhoea diagnosis in these studies [21–23].

Accordingly, the primary aim of this study was to estimate the proportion of genital gonorrhoea cases that were asymptomatic and symptomatic among women attending a sexual health clinic in Melbourne in Australia using nucleic acid amplification test (NAAT). The secondary aim was to describe the time from the last sexual contact to the onset of symptoms and the time to attend health care following the onset of symptoms to determine if these offered any insights into reasons for the recent increases in the infection rates and consequently, to potential public health interventions.

## 2. Methods

### 2.1 Study population

This was a cross-sectional study including all women aged 16 years or above attending the Melbourne Sexual Health Centre (MSHC), Australia, between August 2017 and August 2018. MSHC is a public HIV/STI clinic that offers a range of free clinical services regarding sexual health. MSHC is the largest sexual health clinic in Victoria and provides more than 50 000 clinical consultations a year. For the purposes of this study, only cisgender women were included.

Prior to August 2017, as genital gonorrhoea was considered very uncommon in female attendees, testing was only recommended among women who reported genital symptoms (i.e. presence of discharge or dysuria) or sexual contact with someone diagnosed with gonorrhoea infection as per the Australian STI Management Guidelines [24]. However, MSHC implemented an in-house screening policy in August 2017, and all women attending MSHC, regardless of the presence of symptoms, were offered genital gonorrhoea and chlamydia screening by NAAT using Aptima Combo (AC2) assay (Hologic Panther system; Hologic, San Diego, CA, USA) [25, 26]. All clients attending MSHC were asked if they had any symptoms by the triage nurse and then by the treating clinician. Symptomatic women were examined by the clinician and an endocervical swab was obtained for NAAT and/or culture for *Neisseria gonorrhoeae*. No examination was performed among asymptomatic women [27], but they were asked to self-collect a vaginal swab or first pass urine (FPU) and the specimens were tested by NAAT for *N. gonorrhoeae*. If a swab was collected for culture, it was plated immediately onto modified Thayer-Martin medium for gonorrhoea culture and a smear prepared for Gram stain and microscopy to look for Gram negative diplococci. For individuals who tested positive by NAAT but had no culture performed at the screening visit, a culture test was performed on the subsequent visit before gonorrhoea treatment was administered, to determine the antimicrobial susceptibility profile.

### 2.2 Data collection

We collected data regarding demographic characteristics, recent sexual practices, sex work, self-reported genital symptoms and duration, sexual contact with a person diagnosed with gonorrhoea, investigations performed and laboratory results. For the purpose of this study, we categorised gonorrhoea cases among women into two groups based on their reported symptoms: (i) symptomatic; or (ii) asymptomatic. Symptomatic cases were defined as women who had any genital symptoms on the day of testing. Symptomatic cases included not only women with a characteristic discharge for gonorrhoea (yellow, green or pus-like vaginal discharge) but also reporting other genital symptoms such as dysuria, urethral discomfort, vulvar or vaginal itch, clear abnormal vaginal discharge or lower abdominal pain. Asymptomatic cases were women who reported no genitourinary symptoms on the day of testing.

### 2.3 Data analysis

Descriptive analyses were conducted to summarise the key parameters. The 95% confidence intervals (CIs) for the sample proportions were calculated using Agresti-Coull (adjusted Wald) method. All analyses were conducted using SPSS (version 25, Armonk, NY: IBM Corp). Ethical approval was granted by the Alfred Hospital Ethics Committee, Melbourne, Australia (450/18).

## 3. Results

**Fig. 1.**
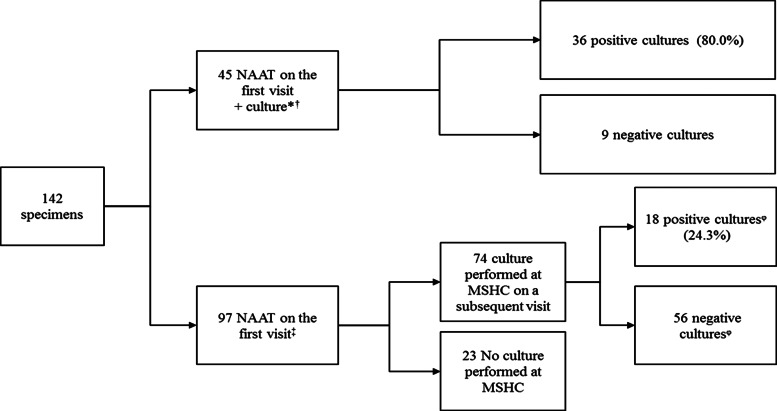
Investigations performed in 142 cases of genital *N. gonorrhoeae* infection among women attending MSHC, 2017–2018. *33 (73.3%) cases were symptomatic, including eight cases with yellow, green or pus-like vaginal discharge, 12 (26.7%) were asymptomatic. ^†^In four cases only culture was performed as it was previously diagnosed outside MSHC. ^‡^56 (57.7%) were asymptomatic, 41 (42.3%) had genital symptoms including eight cases with yellow, green or pus-like vaginal discharge. ^ᵠ^Of the 56 cases with a negative culture in a subsequent visit, 10 (17.9%) had received antibiotic treatment on the first visit (five received treatment for bacterial vaginosis, two for urinary tract infection, two for chlamydia and one for both chlamydia and bacterial vaginosis); among the ones with positive cultures, four (22.2%) had received antibiotic treatment (three for bacterial vaginosis and one for urinary tract infection). NAAT, nucleic acid amplification test; MSHC, Melbourne Sexual Health Centre.

There were 17 258 clinic visits by 9302 individual women attending MSHC during the study period. Genitourinary specimens were obtained (FPU, endocervical or vaginal specimens) in 12 869 (74.6%) clinic visits from 7350 individual women. Genital gonorrhoea was detected in 1.1% (142/12 869) of the clinic visits where women were tested for genital gonorrhoea, corresponding to 142 different gonorrhoea cases in 140 individual women. The investigations performed including NAAT and culture for *N. gonorrhoeae* are shown in Figure 1. The median age of the 142 genital gonorrhoea cases was 28 years [interquartile range (IQR) 24–34], 79 (55.6%) were born overseas and 13 (9.2%) had a previous gonorrhoea diagnosis at the MSHC. The median number of male sexual partners in the preceding three months was 2 (IQR 1–4) and 12 (8.5%) reported having a female sexual partners in the preceding three months. Only 12 (8.5%) reported always using condoms with male partners for sex in the preceding three months, 37 (26.1%) reported having sex overseas in the preceding 12 months and 52 (36.6%) were currently working as sex workers (Table 1).

**Table 1.** Demographic and sexual behaviour characteristics of 142 cases of genital *N. gonorrhoeae* infection among women attending MSHC, 2017–2018

Characteristics	All women (*N* = 142)	Symptomatic women (*N* = 74)	Asymptomatic women (*N* = 68)	*P* value^‡^
Age (years), median (IQR)	28 (24–34)	27 (24–33)	29 (24–36)	0.323
Age (years), *n* (%)				0.556
16–25	55 (38.7)	30 (40.5)	25 (36.8)	
26–35	57 (40.1)	31 (41.9)	26 (38.2)	
≥36	30 (21.1)	13 (17.6)	17 (25.0)	
Country of birth, *n* (%)				0.722
Australia	55 (38.7)	31 (41.9)	24 (35.3)	
Overseas	79 (55.6)	39 (52.7)	40 (58.8)	
No information	8 (5.6)	4 (5.4)	4 (5.9)	
Indigenous status, *n* (%)				0.206
Indigenous origin	2 (1.4)	2 (2.7)	0 (0)	
Non-indigenous origin	103 (72.5)	50 (67.6)	53 (77.9)	
No information	37 (26.1)	22 (29.7)	15 (22.1)	
Number of male sexual partners in the preceding 3 months, median (IQR)*	2 (1–4)	2 (1–3)	2 (1–5)	0.965
Number of male sexual partners in the preceding 3 months, *n* (%)				0.082
≤4	82 (57.7)	49 (66.2)	33 (48.5)	
>4	21 (14.8)	10 (13.5)	11 (16.2)	
No information	39 (27.5)	15 (20.3)	24 (35.3)	
Had a female sexual partner(s) in the preceding 3 months, *n* (%)				0.550
Yes	12 (8.5)	5 (6.8)	7 (10.3)	
No	104 (73.2)	57 (77.0)	47 (69.1)	
Not information	26 (18.3)	12 (16.2)	14 (20.6)	
Condom use with male sexual partners in the preceding 3 months, *n* (%)				0.205
Always	12 (8.5)	5 (6.8)	7 (10.3)	
Not always^†^	88 (62.0)	51 (68.9)	37 (54.4)	
No information	42 (29.6)	18 (24.3)	24(35.3)	
Sexual contact with a man diagnosed with gonorrhoea, *n* (%)				0.650
Yes	25 (17.6)	12 (16.2)	13 (19.1)	
No	117 (82.4)	62 (83.8)	55 (80.9)	
Current sex worker, *n* (%)				0.011
Yes	52 (36.6)	19 (25.7)	33 (48.5)	
No	83 (58.5)	52 (70.3)	31 (45.6)	
No information	7 (4.9)	3 (4.1)	4 (5.9)	
Sex overseas in the preceding 12 months, *n* (%)				0.952
Yes	37 (26.1)	19 (25.7)	18 (26.5)	
No	77 (54.2)	41 (55.4)	36 (52.9)	
No information	28 (19.7)	14 (18.9)	14 (20.6)	

IQR: interquartile range. Data are presented as either *n* (%) or median (IQR).

*The median of male sex partners was calculated among 103 women who reported the number of male partners, excluding the 39 women who decided not to report the number of male partners in the preceding three months.

^†^‘Not always’ was defined as women who sometimes, usually or never used a condom with their male partners in the preceding 3 months.

^‡^Chi-squared test was used to compare categorical variables between symptomatic and asymptomatic women. Mann-Whitney *U* test was used to compare continuous variables between symptomatic and asymptomatic women.

Almost half of the cases (*n* = 68, 47.9%, 95% CI 39.8–56.1%) reported no symptoms (Table 2). Of the 74 cases (52.1%, 95% CI 44.0–60.1%) where women reported any symptom, the most common symptoms reported included a clear abnormal vaginal discharge (*n* = 29, 39.2%), dysuria (*n* = 23, 31.1%) and a change in vaginal discharge to a yellow, green or pus-like vaginal discharge (*n* = 16, 21.6%; 11.3% [95% CI 7.0–17.6%] over the total number of cases). A smaller proportion also reported *Per Vaginal* (PV)/postcoital bleeding (*n* = 9; 12.2%) and pelvic pain (*n* = 8; 10.8%) (Table 3). Among the 142 genital gonorrhoea cases, 18 (12.7%) had concurrent bacterial vaginosis and 11 (7.7%) had pelvic inflammatory disease. Of the 25 (17.6%) cases with sexual contact with a male diagnosed with gonorrhoea, 13 (52.0%) were asymptomatic, and 12 (48.0%) symptomatic, including three cases with yellow, green or pus-like vaginal discharge.

**Table 2.** Clinical characteristics of cases of genital *N. gonorrhoeae* infection among women attending MSHC, 2017–2018

Characteristics	*n/N*	% (95% CI)
**History**		
Genital symptoms reported on day of test		
Symptomatic	74/142	52.1 (44.0–60.1)
Days between last sexual contact and onset of symptoms, N, mean (SD)	10*	7.3 (4.4)
Days between onset of symptoms and presentation to the clinic, N, mean (SD)	60*	12.1 (14.6)
Asymptomatic	68/142	47.9 (39.8–56.1)
**Investigations^†^**		
Gonorrhoea culture positive from genital swab		
Symptomatic	35/64	54.7 (42.6–66.3)
Asymptomatic	19/56	33.9 (22.9–47.0)
Tested positive for genital chlamydia		
Symptomatic	9/69	13.0 (6.8–23.2)
Asymptomatic	9/66	13.6 (7.1–24.2)

CI, confidence interval; SD, standard deviation; NAAT, nucleic acid amplification test.

*10 and 60 women reported date of last sexual contact and date of onset of symptoms, respectively. ^†^The denominator of the percentage is the total of specimens in which the investigation was performed.

**Table 3.** Symptoms present on the day of testing among 74 symptomatic cases of genital *N. gonorrhoeae* infection among women attending MSHC, 2017–2018

Symptoms*	*n*	%
Clear abnormal vaginal discharge	29	39.2%
Dysuria	23	31.1%
Yellow, green or pus-like vaginal discharge	16	21.6%
Urinary frequency/nocturia	11	14.9%
Vaginal odour	10	13.5%
Dyspareunia	10	13.5%
PV bleeding/postcoital bleed	9	12.2%
Vulvar redness	6	8.1%
Vulvar itch	6	8.1%
Hematuria	2	2.7%
Genital ulcer	2	2.7%
Vulvar pain	2	2.7%
Vaginal itch	2	2.7%
Urethral discomfort	1	1.4%
Vaginal pain	1	1.4%
Perineal pain	1	1.4%

*There were cases in which more than one symptom was present

There were 10 symptomatic women who reported the date of last sexual contact, and the mean time between last sexual contact and the onset of symptoms was 7.3 days [standard deviation (SD) 4.4, ranging from 2 to 16 days]. In addition, there were 60 symptomatic women who reported the date of symptom onset, and the mean time between the onset of symptoms and presentation to the clinic was 12.1 days (SD 14.6, ranging from 1 to 90 days) (Table 2). Among these 60 symptomatic women, 22 (36.7%) presented to the clinic up to 5 days after symptoms onset, 16 (26.7%) between 6 and 10 days and 22 (36.7%) after 10 days or more.

Of the 142 cases with genital gonorrhoea, 135 were also tested for genital chlamydia and 18 (13.3%) tested positive for genital chlamydia by NAAT. Among 68 asymptomatic cases, the positivity for genital chlamydia was 13.6% and among symptomatic was 13.0% (Table 2).

## 4. Discussion

In this clinical audit of 142 confirmed genital gonorrhoea cases in women attending a sexual health clinic in Melbourne, we found that 48% of the cases among women did not have any reported genital symptoms on the day of testing, and only an 11% had typical *N. gonorrhoeae* discharge (i.e. yellow, green or pus-like vaginal discharge). This suggests that a substantial proportion of asymptomatic cases would have been missed without screening of asymptomatic women. This result also contrasts with heterosexual men where more than 90% of genital cases are symptomatic [28]. Along similar lines, time from symptoms onset to presentation to the clinic was of 12 days, which could be partially explained by the non-specific symptoms of gonorrhoea. Given the importance of the early detection of infectious cases for effective gonorrhoea control, it is possible that a public health campaign aimed at increasing awareness and knowledge on gonorrhoea, as well as, improving testing of gonorrhoea in symptomatic women and asymptomatic women at risk may be indicated.

There are some limitations in the study that should be noted. First, this study was conducted in one urban major public sexual health clinic in Melbourne that sees only 25% of the cases of gonorrhoea diagnosed in women. Our data may therefore not be representative of presentations to general practice [29]. Women in our study may be biased towards to those with genital symptoms or contacts of infection. Besides that, sex workers may be overrepresented in our study for the mandatory three-monthly HIV/STI screening [30]. Second, recall bias might have occurred when women reported the time between the onset of symptoms and last sexual contact. Moreover, a substantial proportion of data was missing because it was not always systematically collected, hence we could only present the results of the time between last sexual contact and the onset of symptoms when the data is available. Third, sexual health services in Melbourne are quite limited and it is not always possible to see all individuals on the day that they present symptoms [17]. This could have contributed to more than five days elapsed since symptoms onset to presentation to the clinic in almost two-thirds of the cases. It is therefore possible that the time between the onset of symptoms and presentation may be longer in our study than for women attending other services.

There have been very limited studies estimating the proportion of gonorrhoea in women that is asymptomatic and most of these studies were conducted in the 1970s. Asymptomatic screening in women is not currently recommended in many countries such as the USA and UK. In addition, clinical diagnosis of gonorrhoea in symptomatic women is always difficult because the presence of non-specific symptoms (e.g. dysuria, dyspareunia, vulvar itch or pelvic pain) may be confounded by other infections such as bacterial vaginosis, *Chlamydia trachomatis* and *Trichomonas vaginalis*. In our study, we found that 48% of women who tested positive for *N. gonorrhoeae* did not report any genital symptoms on the day of screening. However, McCormack and colleagues reviewed 278 women with gonorrhoea in 1974 in Boston in a hospital setting and they found that only 19% of the cases were asymptomatic [31]. Furthermore, we found that 52% (13/25) of gonorrhoea contacts that tested positive for genital gonorrhoea were asymptomatic. Our previous audit in 2017–18 showed 44% (26/59) of asymptomatic female gonorrhoea contacts tested positive by NAAT at the genital site [32]. However, Pariser and colleagues (1968) found that only 29% of asymptomatic female gonorrhoea contacts were culture-positive for gonorrhoea [33]. The low positivity in Pariser and colleagues’ study may be due to poor sensitivity in culture compared to NAAT. Past studies have shown that the majority of women with gonorrhoea would develop symptoms within 9–11 days after sexual exposure and this is similar to the estimate of 7.3 days in our study [22, 23].

The increase in the rates of gonorrhoea among women in Australia has happened alongside an increase in the incidence of chlamydia and infectious syphilis since the mid-2010s [2, 34, 35]. This has also led to the re-emergence of congenital syphilis in Victoria in 2017, which has not emerged in the state since 2004 [34, 35]. The Australian national surveys have revealed that there were no significant changes in the number of partners and condomless sex among women in Australia between 2001 and 2013 [11, 12]; however, the much of the STI rise occurs after 2013, and there have been very limited behavioural studies examining sexual practices among women in Australia. The rises in gonorrhoea in women in Australia remain unclear and it may be due to other unmeasured factors (e.g. group sex, use of smartphone dating applications, transmission from higher-incidence populations) that may have changed over time. Nevertheless, given the importance of access to health care for the control of STIs, public health campaigns and sexual health educations are important to increase the awareness and recognition of STI-related symptoms. General practitioners should also be informed and trained accordingly to increase the knowledge and awareness of the current STI epidemic, testing and treatment guidelines.

In conclusion, genital gonorrhoea is largely asymptomatic or has an unspecific clinical presentation among women. Testing only women with typical *N. gonorrhoeae* discharge would have missed a considerable number of cases. Further efforts must be made to promote gonorrhoea screening at primary care settings in Victoria, including training on test and treat among general practitioners. Additionally, epidemiological and behavioural research is required to understand the temporal changes in sexual practices among women in Australia and how this could be related to recent increases in the incidence of gonorrhoea.

## Data Availability

All relevant data generated or analysed during this study are included in this published article.

## References

[1] Chow EPF, Grulich AE and Fairley CK (2019) Epidemiology and prevention of sexually transmitted infections in men who have sex with men at risk of HIV. The Lancet HIV Elsevier Ltd 6, e396–e405. 10.1016/S2352-3018(19)30043-8 31006612

[2] Jasek E, (2017) Sexually Transmitted Infections in Melbourne, Australia from 1918 to 2016: nearly a century of data. Communicable Diseases Intelligence Quarterly Report 41, E212–E222. 29720070 10.33321/cdi.2017.41.31

[3] Misson J, (2018) Trends in gonorrhoea infection and overseas sexual contacts among females attending a sexual health centre in Melbourne, Australia, 2008–2015. Communicable Diseases Intelligence 2018, 42. 10.33321/cdi.2018.42.22 30626294

[4] Chow EPF, (2019) Prevalence of genital and oropharyngeal chlamydia and gonorrhoea among female sex workers in Melbourne, Australia, 2015–2017: Need for oropharyngeal testing. Sexually Transmitted Infections 95, 398–401. Published online: 2019. 31113904 10.1136/sextrans-2018-053957

[5] Chow EPF, (2015) Gonorrhoea notifications and nucleic acid amplification testing in a very low-prevalence Australian female population. Medical Journal of Australia 202, 321–323. Published online: 2015. 25832159 10.5694/mja14.00780

[6] Public Health England (2019) Sexually transmitted infections and chlamydia screening in England, 2018. Health Protection Report 13, 19, Published online: 2019.

[7] Sentís A, (2019) Sexually transmitted infections in young people and factors associated with HIV coinfection: An observational study in a large city. BMJ Open 9, e027245, Published online: 2019. 10.1136/bmjopen-2018-027245 PMC6502227 31061051

[8] European Centre for Disease Prevention and Control (2019) Gonorrhoea. In: ECDC. Annual epidemiological report for 2017. Stockholm: ECDC.

[9] Centers for Disease Control. Sexually Transmitted Disease Surveillance 2018. Centre of Disease Control and Prevention 2018. Published online: 2018. doi: 10.1016/j.molmet.2013.04.004.

[10] Savage EJ, (2012) Rapid increase in gonorrhoea and syphilis diagnoses in England in 2011. Eurosurveillance 17, 20224. doi: 10.2807/ese.17.29.20224-en. 22835469

[11] Phillips TR, (2019) Risk factors for urethral gonorrhoea infection among heterosexual males in Melbourne, Australia: 2007–17. Sexual Health 16, 508–513. Published online: 2019. 31203836 10.1071/SH19027

[12] Rissel C, (2014) Heterosexual experience and recent heterosexual encounters among Australian adults: The second Australian study of health and relationships. Sexual Health 11, 416–426. Published online: 2014. 25376995 10.1071/SH14105

[13] De Visser RO, (2003) Sex in Australia: Heterosexual experience and recent heterosexual encounters among a representative sample of adults. Australian and New Zealand Journal of Public Health 27, 146–154. Published online: 2003. 14696705 10.1111/j.1467-842x.2003.tb00802.x

[14] Aung ET, (2019) International travel as risk factor for Chlamydia trachomatis infections among young heterosexuals attending a sexual health clinic in Melbourne, Australia, 2007 to 2017. Eurosurveillance 24, 1900219. Published online: 2019. 10.2807/1560-7917.ES.2019.24.44.1900219 PMC6836681 31690365

[15] Cabada MM, (2009) High prevalence of sexually transmitted infections among young peruvians who have sexual intercourse with foreign travelers in Cuzco. Journal of Travel Medicine 16, 299–303. Publishedo online: 2009. 19796098 10.1111/j.1708-8305.2009.00324.x

[16] Sundbeck M, Agardh A and Östergren PO. (2017) Travel abroad increases sexual health risk-taking among Swedish youth: A population-based study using a case-crossover strategy. Global Health Action 10, 1330511. Published online: 2017. 28598729 10.1080/16549716.2017.1330511 PMC5496094

[17] Needleman R, (2018) Access to sexual health services after the rapid roll out of the launch of pre-exposure prophylaxis for HIV in Melbourne, Australia: A retrospective cross-sectional analysis. Sexual Health 15, 528–532. Published online:2018. 29973331 10.1071/SH17182

[18] Dyer C. (2014) Doctors warn local authorities about putting sexual health services out to tender. BMJ *(*Clinical research ed.) 348, f7715. Published online: 2014. 10.1136/bmj.f7715 24385552

[19] Foley E, (2017) Inequalities in access to genitourinary medicine clinics in the UK: Results from a mystery shopper survey. Sexually Transmitted Infections 93, 472–475. Published online: 2017. 28377420 10.1136/sextrans-2016-052882

[20] Walker CK and Sweet RL (2011) Gonorrhea infection in women: Prevalence, effects, screening, and management. International Journal of Women's Health 3, 197–206. 10.2147/IJWH.S13427 PMC3150204 21845064

[21] Pedersen A and Bonin P (1971) Screening females for asymptomatic gonorrhea infection. Northwest Med 70, 255–61. 5550113

[22] Platt R, Rice PA and McCormack WM (1983) Risk of Acquiring Gonorrhea and Prevalence of Abnormal Adnexal Findings Among Women Recently Exposed to Gonorrhea. JAMA: The Journal of the American Medical Association 250, 3205–3209. Published online: 1983. 6417362

[23] Wallin J (1975) Gonorrhoea in 1972. A 1 year study of patients attending the VD unit in Uppsala. British Journal of Venereal Diseases 51, 41. 1125748 10.1136/sti.51.1.41 PMC1045109

[24] The Australian Government (2019) Department of Health. Australian STI management guidelines for use in primary care. Gonorrhoea. (http://www.sti.guidelines.org.au/sexually-transmissible-infections/gonorrhoea#diagnosis). Accessed 14 February 2020.

[25] Johnson RE, (2002) Screening tests to detect Chlamydia trachomatis and Neisseria gonorrhoeae infections--2002. MMWR. Recommendations and Reports : Morbidity and Mortality Weekly Report. Recommendations and Reports/Centers for Disease Control 51, 1–38. doi: 10.1097/00019048-200206000-00018. 12418541

[26] CDC (Centers for Disease Control and Prevention), (2014) Recommendations for the Laboratory-Based Detection of *Chlamydia trachomatis* and *Neisseria gonorrhoeae* - 2014. Morbidity and Mortality Weekly Report 63, 1–19. 24622331 PMC4047970

[27] Lee DM, (2006) Is routine vaginal examination necessary for asymptomatic women attending sexual health services? International Journal of STD and AIDS 17, 631–632. Published online: 2006. 16942656 10.1258/095646206778113069

[28] Martín-Sánchez M, (2020) Clinical presentation of asymptomatic and symptomatic heterosexual men who tested positive for urethral gonorrhoea at a sexual health clinic in Melbourne, Australia. BMC Infect Dis 20, 486. doi: 10.1186/s12879-020-05197-y. 32641070 PMC7346512

[29] Williamson DA, (2019) Bridging of Neisseria gonorrhoeae lineages across sexual networks in the HIV pre-exposure prophylaxis era. Nature Communications *Springer US* 10, 1–10. Published online 2019. 10.1038/s41467-019-12053-4 PMC6728426 31488838

[30] Chow EPF, (2014) Testing commercial sex workers for sexually transmitted infections in Victoria, Australia: an evaluation of the impact of reducing the frequency of testing. PLoS One 9, e103081. Published online: 2014. 25048817 10.1371/journal.pone.0103081 PMC4105494

[31] McCormack WM, (1977) Clinical spectrum of gonococcal infection in women. The Lancet 309, 1182–1185. 10.1016/s0140-6736(77)92720-9 68279

[32] Chow EPF, (2019) Oropharyngeal and Genital Gonorrhea Infections Among Women and Heterosexual Men Reporting Sexual Contact With Partners With Gonorrhea: Implication for Oropharyngeal Testing of Heterosexual Gonorrhea Contacts. Sexually transmitted diseases 46, 743–747. Published online: 2019. 31517767 10.1097/OLQ.0000000000001068

[33] Pariser H and Farmer AD (1968) Diagnosis of gonorrhea in the asymptomatic female: Comparison of slide and culture technics. Southern Medical Journal 61, 505–506. 4967597 10.1097/00007611-196805000-00013

[34] Kirby Institute (2018) Annual surveillance report 2018 HIV, viral hepatitis and sexually transmissible infections in Australia. Available at: https://kirby.unsw.edu.au/sites/default/files/kirby/report/KI_Annual-Surveillance-Report-2018_0.pdf.

[35] Department of Health & Human Services, State Government of Victoria (2019) Syphilis cases continue to rise in Victoria in both men and women. [online] Available at: https://www2.health.vic.gov.au/about/news-and-events/healthalerts/rising-syphilis-cases-.

